# Influence of socio-economic and demographic factors in determining breathing patterns: a pilot study

**DOI:** 10.1016/S1808-8694(15)31179-4

**Published:** 2015-10-19

**Authors:** Valdenice Aparecida de Menezes, Rossana Barbosa Leal, Marcela Motta Moura, Ana Flávia Granville-Garcia

**Affiliations:** 1 PhD, Professor - University of Pernanbuco; 2 M.S. in Odontopediatrics, PhD - Dental School of Pernambuco; 3 Dentistry student - Dental school of Caruaru; 4 PhD, Professor - Dental School of /ASCES; Faculdade de Odontologia de Pernambuco/UPE Faculdade de Odontologia de Caruaru/ASCES

**Keywords:** breathing problems, socio-economic factor, oral breathing

## Abstract

Breathing represents one of the vital functions of the organism, and its unbalance causes some series of alterations in several organs and systems.

**Aim:**

Verify the influence of socio-economic and demographic factors in determining breathing patterns. Study design: cross-sectional.

**Materials and Methods:**

there were 143 students in the sample, with ages ranging from 9 and 10 years, from two schools, public and private, in the city of Recife, Pernambuco. Breathing patterns were established through two tests: Glatzel Plate (Steam) and water time in the mouth. Socio-economic factors were evaluated through questionnaires with nine questions each. Statistics were carried out by means of the Chi-Squared test or Fisher's Exact test and the significance level used was of 5%.

**Results:**

Oral breathing prevalence was of 55.2%, higher among females (57.7%) and in public schools (67.2%). Lack of medical care (62%), less use of medications (56.6%), parents with educational levels lower than high school, divorced parents (66%), students that do not live with their parents (68.7%) and homes with only one room (72%), in all of those situations, oral breathing signs were more prevalent. Only school type had significant association with the breathing pattern.

**Conclusion:**

High levels of oral breathing without differences concerning gender and age. With the exception of school type, there was no association between breathing pattern and socio-economic factors.

## INTRODUCTION

Breathing is vital; it brings oxygen to the tissues and removes carbon dioxide. When the air inhaled is not properly prepared to reach the lungs, it changes pulmonary mechanisms with consequent inadequate body oxygenation.^1^

For nasal breathing to occur, it is necessary to have functional and anatomical integrity of the airways. A simple mechanical obstruction blocking the air passage is enough for the individual to change his breathing pattern in order to keep his vital functions^2^, thus, oral breathing is considered a supplemental or pathologic breathing.

The disorders caused by a temporary replacement of nasal breathing pattern are overcome by reestablishing proper breathing. The continuity of such disordered breathing may alter mastication, deglution, respiration and phonation, which will later influence the growth and development of systems^3^, and will also change the balance of postural and thoracic muscle forces.^4^,^5^

Most of the times, the oral breathing syndrome is associated with nasal obstruction, because of anatomical or iatrogenic predisposition, however it can be due to deleterious habits such as sucking the thumb or prolonged use of a pacifier^6^.

Considering that respiratory disorders are usually problems of multifactorial character, it is difficult to define clearly the etiology of oral breathing^7^. During childhood, any disease, accident, allergy or cold symptoms may obstruct the upper airway and, with time, lead the child to breathe through the mouth. Breastfeeding is the ideal method to avoid it, because alternative feeding and early weaning predispose the child to developing allergies that will change the respiratory pattern^8^ and, consequently, the occurrence of deleterious oral habits^9^, ^10^, ^11^.

Currently, oral breathing is considered a Public Health Problem and, because of its complexity, many fields in health care have shown a growing interest in the problems caused by such syndrome, because they may affect general health and the individual's life quality^12^, ^13^, ^14^, ^15^ and because of the limitations and complications caused by this pathology^9^,^16^,^17^.

Based on this principle, it is fundamental to diagnose and refer the patient to multidisciplinary treatment as early as possible, while there are no bone deformities on the face, cardiorespiratory, immunological and behavioral changes18. However, the difficulty of access to public health care services and the lack of knowledge of the population about the sequelae caused by the pathology itself may play important roles in this context.

Aware of such conditions, the goal of the present investigation is to check the influence of socioeconomic and demographic factors in determining the respiratory pattern of children from the public and private health care system of the city of Recife-PE.

## MATERIALS AND METHODS

This was a pilot, cross-sectional, descriptive study, in which we assessed 143 children and adolescents with ages varying between 9 ad 10 years, of both genders, properly enrolled in two schools in the metropolitan region of Recife/PE, one private and one public.

Clinical diagnosis regarding breathing pattern was considered in two tests: Test 1 was carried out with the metal plate (Glatzel) to check the presence of the steam distribution (superior /inferior/both) caused by breathing and Test 2, to assess how long water remained in the mouth with the lips touching and without swallowing for 3 minutes (timed), observing through the labial commissure if there was any effort during this period^14^.

For statistical analysis purposes, we considered as oral breathers those patients classified as such in both tests that were carried out, in other words, that had steam in the inferior or inferior/superior portions of the Glatzel metal plate and kept the water in their mouths for three minutes.

In order to check the level of coincidence inter-examiners as to breathing pattern diagnosis we obtained the Kappa coincidence index, of which value was 0.85.

The students were randomly selected, by a lottery, between May and June of 2006. In order to assess socioeconomic factors, the students were interviewed after proper and signed authorization by the parents/guardians, making use of a previously created form with questions dealing with the following aspects: medical care, use of medication, who they live with, family structure (marital status of the parents), number of rooms in the house where they lived, number of siblings, maternal and paternal education, type of work the mother did.

This study was approved by the Ethics and Research Committee of the University of Pernambuco, and followed the standards established by Resolution 196/96 from the National Committee of Ethics and Research of the National Health Council.

For data analysis we obtained absolute and percentage distributions for the variables in nominal scale and also statistic measures such as: minimum, maximum, mean, median, standard deviation and variation coefficient for age (descriptive statistics techniques), and we also used the following statistical tests: chi-squared for proportions equality or Fisher's Exact Test when it was not possible to use the Chi-Squared.

In order to determine the impact of independent variables on the dependent variable (student with an oral breathing pattern), we adjusted a logistics regression model considering the significant variables with the dependent variable at the 20% level (p < 0.20) in the bi-varied study.

Data were keyed in the Excel spreadsheet and we used the SAS (Statistical Analysis System), version 8.0. statistics software for data analysis. We used 5.0% as level of significance in the statistical tests.

## RESULTS

Of the 143 students who took part in the study, 79 (55.2%) came from the private school and 64 (44.8%) came fro the public school. As to gender, 54.5% were males and 45.5%, were females. Most students did not use medications. Among the ones who used it, most of them came from the private school (21.8% x 4.7%) and of those, most of them (93.6% x 29.7%) had medical insurance.

The rate of divorced parents was higher among students from the public school (50.0% x 22.7%) and the contrary happened with the rate of married parents, which was higher among students from the private school (73.4% x 43.7%); as to the issue of who the children lived with, more children answered they lived with both parents among those from the private school (75.9% x 39.1%) and who lived with the mother only was higher among children from the public school (40.6% x 20.3%); a large number of people (6 or more) who lived at home tended to be higher among students from the public school (34.4% x 17.9%). The number of rooms in the house (3 or more) tended to be higher in the homes of students from the private school (78.4% x 21.6%). Except for the order of childbirth, we can see significant differences between the two types of school at the significance level considered for each one of the variables we analyzed. Parents' educational level was higher among students from the private school, since 68.4% had higher education and 71.7% of the parents of children in the public school hadn't even finished basic education; As to the mothers' level of education, the rate of mothers who did not complete basic education was 66.7% in the public school and 72.7% of mothers with higher education in the private school children. The number of mothers who had a job was also higher among children from the private school (73.4% x 49.2%). Of the mothers who worked (at home or had jobs) most were on wages, and this rate was higher among mothers of children from the public school (79.4% x 65.0%). Except for the profession (self-employed or employed by third parties) we see a significant difference between the parents of children from the two types of school for the other variables analyzed.

As far as the respiratory pattern diagnostic is concerned (Table 1) we noticed that the number of oral breathing students in Test 1 (< 3 minutes with water in their mouths), was higher among those from the public school (68.7%), being statistically significant. In Test 2, we noticed that most students (53.8%) also were oral breathers (vapor inferior/both), and this percentage was similarly higher among students from the public school (65.6%).

**Table 1:** Breathing pattern evaluation according to the results from the tests of water dwelling time in the mouth (in minutes) and the steam in the mirror, according to the type of school.

Type of school							
Variables	Private		Public		Group Total		P value
	N	%	n	%	N	%	
• Breathing Pattern Time (minutes)							
< 3 (RO)(2)	37	46.8	44	68.7	81	56.6	p(1) = 0.0086*
> = 3 (RN)(2)	42	53.2	20	31.3	62	43.4	
TOTAL	79	100.0	64	100.0	143	100.0	
• Steam in the mirror							
Superior (RN)(2)	43	54.4	21	32.8	64	44.8	p(1) = 0.0298*
Inferior (RO)(2)	1	1.3	1	1.6	2	1.4	
Both (RO) (2)	35	44.3	42	65.6	77	53.8	
TOTAL	79	100.0	64	100.0	143	100.0	

(*) Significant difference at 5.0%.

(1) Through the Pearson's chi-squared test.

(2) Results regarding breathing type - RO oral breathing

RN nasal breathing

On Tables 2 through 5, we show the results from the breathing pattern assessment according to the variables: type of school, gender, age range, medical care, use of medication, family structure with parents, who they lived with, birth order, number of people living in the house, number of rooms, father's education, mother's education, mother's profession.

**Table 2:** Breathing pattern assessment concerning the following variables: gender and age:

Breathing pattern							
Variables	Nasal		Oral		Group Total		P value
	N	%	N	%	N	%	
• School type							
Private	43	54.4	36	45.6	79	100.0	p(1) = 0.0097*
Public	21	32.8	43	67.2	64	100.0	
TOTAL	64	44.8	79	55.2	143	100.0	
• Gender							
Males	31	47.7	34	52.3	65	100.0	p(1) = 0.5191
Females	33	42.3	45	57.7	78	100.0	
TOTAL	64	100.0	79	100.0	143	100.0	
• Age range (in years)							
9	32	45.1	39	54.9	71	100.0	p(1) = 0.9400
10	32	44.4	40	55.6	72	100.0	
TOTAL	64	100.0	79	100.0	143	100.0	

(*) Significant difference at 5.0%.

(1) Through the Pearson's Chi-Squared Test.

The students diagnosed as oral breathers were those classified as such in the two tests carried out. After data crossover: of the 143 students, 64 (44.8%) were diagnosed as nasal breathers and 79 (55.2%) as oral breathers, and this latter figure was higher among students from the public school (67.2%) with statistically significant difference (Table 2). Nonetheless, as far as gender is concerned, there was no statistical difference.

The rate of oral breathing students was of 9.8%, higher among those who did not have medical care (62.0%), however, as far as medication use is concerned, the data was similar (Table 3).

**Table 3:** Variables evaluation: medical care and the use of medication according to breathing pattern.

Breathing pattern							
Variables	Nasal		Oral		TOTAL		P value
	n	%	n	%	n	%	
• Medical care							
Yes	44	47.8	48	52.2	92	100.0	p(1) = 0.2603
No	19	38.0	31	62.0	50	100.0	
Group Total(2)	63	44.4	79	55.6	142	100.0	
(2) - We did not have this information for one of the students.							
• Use of medication							
Yes	10	50.0	10	50.0	20	100.0	p(1) = 0.5843
No	53	43.4	69	56.6	122	100.0	
Group Total(2)	63	44.4	79	55.6	142	100.0	
(2) - We did not have this information for one of the students.							

(1) - By means of the Pearson's Chi-Squared.

Of the data regarding family structure, we highlight that the percentage of oral breathers was higher among those from divorced parents (66.0%) and among those who did not live with the mother or with the father and the mother (68.7%). It was inversely proportional to the number of rooms in the house (72.0% for those who had only one room), without significant association (Table 4).

**Table 4:** Variables evaluation: family structure, who they live with, birth order, number of people living in the house and how many rooms are there, according to breathing pattern.

Breathing pattern							
Variables	Nasal		Oral		TOTAL		P value
	n	%	n	%	n	%	
• Family structure - parents							
Divorced	17	34.0	33	66.0	50	100.0	p(1) = 0.1435
Married	43	50.0	43	50.0	86	100.0	
Widower / widow	4	57.1	3	42.9	7	100.0	
Total Group	64	44.8	79	55.2	143	100.0	
• Who they lived with With both (father and mother)							
	42	49.4	43	50.6	85	100.0	p(2) = 0.3268
The mother only	17	40.5	25	59.5	42	100.0	
Another situation (grandparents. uncles/aunts)	5	31.3	11	68.7	16	100.0	
Total Group	64	44.8	79	55.2	143	100.0	
• Birth order							
1st	28	45.2	34	54.8	62	100.0	p(2) = 0.6084
2nd	16	39.0	25	61.0	41	100.0	
3rd or more	20	50.0	20	50.0	40	100.0	
Total Group	64	44.8	79	55.2	143	100.0	
• Number of people living in the house							
2 or 3	12	52.2	11	47.8	23	100.0	p(2) = 0.5075
4	16	41.0	23	59.0	39	100.0	
5	17	38.6	27	61.4	44	100.0	
6 or more	19	52.8	17	47.2	36	100.0	
Total Group(3)	64	45.1	78	54.9	142	100.0	
(3) - We did not have this information for one of the students.							
• Number of rooms/bedrooms in the house							
1	7	28.0	18	72.0	25	100.0	p(2) = 0.1022
2	12	40.0	18	60.0	30	100.0	
3 or more	45	51.1	43	48.9	88	100.0	
Total Group	64	44.8	79	55.2	143	100.0	

(*) - Significant difference at 5.0%.

(1) - By means of Fisher's Exact Test.

(2) - By means of the Pearson's Chi-Squared test.

On Table 5, we highlight that the higher the educational level of the father and the mother, the lower is the oral breathing rate, however without significant association.

**Table 5:** Variables evaluation: Educational level of father and mother, mother with a job and profession, according to breathing pattern.

Breathing pattern							
Variables	Nasal		Oral		TOTAL		P value
	n	%	n	%	n	%	
• Father's Education							
Did not finish basic education	17	34.0	33	66.0	50	100.0	p(1) = 0.1095
Did not finish intermediate education	7	38.9	11	61.1	18	100.0	
With Intermediate/higher education	36	52.9	32	47.1	68	100.0	
Total Group(3)	60	44.1	76	55.9	136	100.0	
(3) - We did not have this information for seven of the students.							
• Mother's Education							
Did not finish basic education	18	35.3	33	64.7	51	100.0	p(1) = 0.2190
Did not finish intermediate education	11	45.8	13	54.2	24	100.0	
With Intermediate/higher education	32	51.6	30	48.4	62	100.0	
Total Group (3)	61	44.5	76	55.5	137	100.0	
(3) - We did not have this information for six of the students.							
• Mother has a job							
Yes	41	45.6	47	53.4	88	100.0	p(1) = 0.6226
No	22	42.3	30	56.7	30	100.0	
Total Group(3)	63	45.0	77	55.0	140	100.0	
(3) - We did not have this information for three of the students							
• Profession							
Self-employed	13	46.4	15	53.6	18	100.0	p(1) = 0.9309
Employed	30	45.4	36	54.6	66	100.0	
Total Group(3)	43	45.7	51	54.3	94	100.0	
(3) - These data regard 88 mothers who had a job out of the home and 6 who worked as self-employed at home.							

(*) - Significant difference at 5.0%.

(1) - By means of the Pearson's Chi-squared test

(2) - By means of Fisher's exact test.

As we adjusted the logistics regression model for oral prevalence we considered the following independent variables: school type, family structure, number of rooms in the house and parents' education; such variables did have significant association with the breathing pattern at the 20% level. In adjusting the single significant variable at 5%, it was school type, and for such reason we do not present the proposed model because in the present case the problem is based on a bi-varied analysis.

## DISCUSSION

The oral breathing syndrome is increasingly gaining importance in the literature because it is a public health problem^19^, since its long duration can cause^14^,^15^ a series of consequences to the growth and development of the individual, impacting the physical, psychological and social aspects. Thus, health policies that add to early prevention and treatment strategies must be implemented within a multidisciplinary philosophy in order to try to avoid symptomatic treatment^20^,^3^,^15^.

In the present investigation, in order to diagnose breathing pattern, 2 tests were carried out, and their results are different and complementary to each other^21^,^22^: the Glatzel's metal plate^23^,^24^ associated with water dwelling in the mouth for 3 minutes^12^. These tests are fundamental, especially when one tries to analyze exclusive oral breathers, who are identified through the vapor distribution test, which helps differentiate individuals with mixed breathing.

In so far as the importance of the aforementioned tests, we stress that the water dwelling in the mouth test, although enough by itself to diagnose the breathing pattern when used alone, in other words, in cases in which it is not necessary to pick up the exclusive oral breather one must use the 3 minute water test, thus avoiding biased results which can be misinterpreted. The use of shorter periods, 1 to 2 minutes, for example^12^,^24^ can underestimate the prevalence of this problem in the population.

Oral breathing prevalence in the sample studied was of 55.2%, similar to other epidemiological studies that found rates between 45% and 53.3%^21^,^25^, ^26^, ^27^. Nonetheless, the literature is in disagreement showing higher rates (66.3% and 77.7%)^28^,^7^, as well as lower ones (5% to 30%)^29^, ^30^, ^31^, ^32^, ^33^. These differences can be explained by the methodologies used in other investigations, especially in regards of the diagnostic criteria.

As far as gender is concerned, the literature reports that oral breathing is more frequent among males^21^, nonetheless, we found a slight prevalence of oral breathers among females (57.7%) when compared to males (52.3%), however, without significant difference. Such data corroborate another study in which there was a slight difference in this variable^34^.

As we analyze the socioeconomic impact on breathing pattern, we noticed that the oral breathing prevalence was significantly higher among children from the public school (67.2%) when compared to those from the private school (45.6%). Similar data were found in another study about oral sucking habits in which the oral breathing prevalence was of 77.7% in an underprivileged population^7^.

Having in mind that the low-income population is the one bearing the greatest risk because it involves economic and demographic factors^35^, it is without doubt that the prevalence of respiratory disease in children would be reduced if they had better housing and less people living in the same house^36^,^37^. This last data corroborates the findings of this study, because 72% of the children who lived in houses with one room only were oral breathers, and this percentage fell as the number of rooms in the house increased (Table 4).

In order to check other aspects associated with socioeconomic conditions, we asked the children about health insurance and the use of medication (allergy drugs), and we noticed that the majority (93.6%) of the private school children had medical insurance and their frequency of medication use was higher (21.8%); differently from the public school children, among whom only 29.7% had medical insurance and only 4.7% used medication.

When we evaluated whether medical care and medication use were associated with breathing pattern, we noticed that not having medical insurance was associated with a higher percentage of children with oral breathing (62.0%), although such variable, as well as that of medication use were not significant (Table 2). These data call our attention, especially in our country, because precarious socioeconomic conditions worsen diseases. Because of difficult access to specialists in the public health care system, the low-income population not always get the medical care they need, thus leaving numerous respiratory problems without proper care^33^. Moreover, individuals with less means to acquire medication, allocated in public schools, could be those that need it the most.

Individuals with respiratory disorders are more prone to having repetitive episodes of colds, spasmodic cough and hoarseness. They may also develop other disorders, such as: cranial-facial deformities, malocclusion, dry lips, sleepy face, spots around the eyes, speech disorders, postural and gait changes, which all interfere in school performance, professional performance and social relations^38^,^39^,^21^,^22^. Moreover, they have a greater tendency in developing caries and periodontal problems because of the drying in their oral cavities, which without proper lubrication becomes more prone to bacteria and biofilm^40^.

Analyzing family data by type of school, we notice that the percentage of married parents was higher among students from the private school (73.4%) and that of divorced parents was higher among children who went to the public school (50.0%); about who they lived with, the highest percentage of children living with both parents was found among those from the private school system (75.9%) and living with the mother only was mostly found among those from the public school (40.6%); a greater number of people living in the same house was higher with the public school children, while the largest number of rooms in the house was higher among those from the private schools, with significant differences. Nonetheless, at a 5% significance level, there was no association between these variables and breathing pattern (Table 4).

As to the number of people living in the house, results vary when compared to those from other studies, in which the large number of individuals per bedroom was associated with respiratory diseases, especially in relation to the number of people sharing the bedroom with the child^41^,^36^,^37^. Unfavorable dwelling conditions represent an important risk factor for respiratory diseases^42^.

Insofar as social indicators are concerned, we noticed that the lower education of fathers (71.7% and 66.7%) and of mothers who did not have a job was significantly higher among children from public schools. When we assessed these factors in relation to breathing pattern, we noticed that there was no association among any of the variables studied. (Table 5). Nonetheless, the prevalence of oral breathing was higher among children of parents who did not complete basic education.

The occupation and education of those responsible for the family have been associated with respiratory disorders in many studies, especially mothers' education, mentioned as an important factor that determines acute respiratory disorders^36^,^37^. Thus, the more unfavorable the socioeconomic situation, the greater the prevalence and severity of disorders. However, respiratory disorders may vary from mild allergy processes, all the way to severe situations such as sleep apnea; problems which the socioeconomic conditions may have different outcomes^43^.

In this context, it is clear that the oral breathing syndrome is a problem that affects all social layers of the population, where socioeconomic factors play a very important role, starting on prevention, with breastfeeding^44^,^45^ which contributes to the child's immunization and the adoption of a proper respiratory pattern, all the way to creating opportunities for early diagnosis by providing access to information and health care.

Notwithstanding, it is paramount to train multidisciplinary teams and helpers about the Oral Breathing Syndrome, aiming at obtaining a precise diagnosis and to refer the child to full treatment, to improve the life quality of all those who need it.

## CONCLUSIONS

1. Oral breathing prevalence was high in the population studied, with no difference gender wise.
2. There were no associations between the socioeconomic variables evaluated and the breathing pattern, except with school type.
3. Oral breathing was more prevalent among public school children.

## References

[1] Sá Filho FPG (2004). Fisiologia Oral..

[2] Nicolósi R. Respiração bucal [serial online] 2003 [cited 2003 Mar 24]. Available from: URL: http://www.geocities.com/fonobr/respiracao.htm.

[3] Ribeiro A (1998). Respiração bucal e alterações esqueléticas e dentárias [monografia]..

[4] Spinelli MLM, Casanova PC. Respiração bucal. [serial online] 2002fev [cited 2006 Set24]. Availablefrom: URL:http://www.odontologia.com. br/imprimir.asp?id=224&idesp=14.

[5] Carvalho GD, Brandão G, Vinha PP (2002). Os respiradores bucais e as desordens buco-dentais. In: Odontopediatria: prevenção.

[6] Miranda PP, Mashuda SYK, Periotto MC, Araújo RJH (2002). Enfoque multidisciplinar na síndrome do respirador bucal.. Rev. APCD.

[7] Cavassani VGS, Ribeiro SG, Nemr NK, Greco AM, Köhle LehnCN (2003). Hábitos orais de sucção: estudo piloto em população de baixa renda.. Rev. Bras. Otorrinolaringol.

[8] Lusvarghi L (1999). Identificando o respirador bucal.. Revista da APCD.

[9] Carvalho GD (1996). Síndrome do respirador bucal ou insuficiente respirador nasal.. Revista Secretários de Saúde.

[10] CARVALHO, GD. Alterações comportamentais comuns na S.R. B., 2000 disponível em:http://www.ceaodontofono.com.br/artigos/art/2000/mar00.htm. Acessado em: 24 de setembro de 2006.

[11] Granville-Garcia AF, Menezes VA, Lima N, Zirmeman M (2002). Importância da Amamentação: Uma Visão Odontológica.. Arquivos em Odontologia.

[12] Paiva JB (1999). Identificando o respirador oral.. Rev. APCD.

[13] Martinez JE, Barauna Filho IS, Kubokawa K, Pedreira IS, Machado L. A, Cevasco G (1998). Análise crítica dos parâmetros de qualidade de vida de pacientes com fibromialgia.. Acta fisiátrica.

[14] Jorge EP (2001). Estudo da resistência nasal em pacientes com má oclusão de classe II divisão 1^a^ de Angle, utilizando a rinomanometria anterior ativa.. R Dental Press Ortodon Ortop Facial.

[15] Leal RB. Elaboração e validação de um instrumento para avaliar a qualidade de vida do respirador oral [dissertação]. Camaragibe (PE): Universidade de Pernambuco.;2004.

[16] Coimbra C. O tratamento da respiração bucal. [serial online] 2002 [cited 2003 Mar 24]. Available from: URL: http://www.jfservice.com.br/viver/arquivo/dicas/2002/10/17-Cal/.

[17] Alvarenga AL, Pádua IPM, Silveira IA (2003). O respirador bucal.. Pro Homine.

[19] Carvalho GB. S.O.S Respirador Bucal- obstáculos nas diferentes estruturas dificultando ou impedindo o livre processo respiratório [serial online] 1999 out [cited 2003Out15]. Availablefrom: URL:http://www.ceaodontofono.com.br/publicacoes/out99_respirador.html.

[20] Parizotto SPCAL, Nardão GT, Rodrigues CRMD (2002). Atuação multidisciplinar frente ao paciente portador da síndrome da respiração bucal.. JBC.

[21] Pessôa RS, Pontes RMES (2005). Prevalência e fatores associados à respiração oral em escolares participantes do projeto Santo Amaro-Recife, 2005 [monografia]..

[22] Menezes VA, Leal RB, Pessoa RS, Pontes RMES (2006). Prevalência e fatores associados à respiração oral em escolares participantes do projeto Santo Amaro-Recife, 2005.. Rev. Bras. Otorrinolaringol.

[23] Padovan BAE (1976). Reeducação mioterápica nas pressões atípicas de língua: diagnóstico e terapêutica.. Ortodontia.

[24] Rakoski T, Schimith GPF, T. M Graber, B Newmann (1987). Aparelhos ortodônticos removíveis..

[25] Meirelles RC (2001). Obstrução nasal e nódulos vocais.. Rev. Bras. Otorrinolaringol.

[26] Andrade EMF, Ferreira FAC, Macedo AM, Scavoni Júnior H (2005). A influência do padrão respiratório no padrão craniofacial e a morfologia mandibular: estudo cefalométrico.. Ortodontia.

[27] Santos JA, Barros LF, Azevedo MFA (2006). Avaliação da qualidade de vida e do padrão de respiração em crianças portadoras de asma. [monografia]..

[28] Lessa FCR, Enoki C, Feres MFN, Valera FCP, Lima WTA, Matsumoto MAN (2005). Breathing mode influence in craniofacial development.. Rev. Bras. Otorrinolaringol..

[29] Rojas VR, Baez J, Rojas R (2001). Prevalência de malos hábitos orales y respiración bucal em niños de 5 a 17 años del área de Santiago Centro.. Rev. Fac. Odontol. Univ. Chile.

[30] Wendel A, Albejant M F C, Coladeti A PFP, Assencio-Ferreira VJ (2002). Relação causal entre a respiração oral e dificuldades na aprendizagem.. Rev. CEFAC.

[31] Kharbanda OP, Sidhu S S, Sundaram KR, Shukla D Kd (2003). Oral habits in school going children of Delhia prevalence study.. J. Indian Soc. Pedo Prev. Dent..

[32] Montiel JME (2004). Frecuencia de maloclusiones y su asociación com hábitos perniciosos em uma población de niños mexicanos de 6 a 12 años de edad.. Rev ADM.

[33] Araújo AS, Moura JR, Camargo A (2004). Principais sintomas otorrinolaringológicas em escolares.. Arq. Int. Otorrinolaringol..

[34] Polanco CMS, Saldña AR, Yañez EER, Araújo RC (2005). Ortodoncia.

[35] Markovitz BP, Andresen EM (2006). Lack of insurance coverage and urgent care us for asthma: A retrospective cohort study.. BMC Public Health.

[36] Prietsch SOM, Fischer GB, Cesar JA, Fabris AR, Mehanna H, Ferreira THP (2002). Doença aguda das vias aéreas inferiores em menores de cinco anos: influência do ambiente doméstico e do tabagismo materno.. J. Pediatr.

[37] Prietsch SOM, Fischer GB, César JA, Lempek BS, Barbosa LV, Zogbi L (2003). Doença respiratória em menores de 5 anos no sul do Brasil:influência do ambiente doméstico. Rev.. Panam Salud Publica.

[38] Queluz DP, Gimenez CMM (2000). A síndrome do respirador bucal.. Revista do CROMG.

[39] Amaral CSF, Martins ER, Rios JBM (2002). A respiração bucal e o desenvolvimento do complexo dentofacial.. Rev. Bras. Alerg. Imunopatol..

[40] Koga CY, Unterkircher C S, Fantinato V, Watanabe H, Jorge A O C (1996). Influencia da síndrome do respirador bucal na presença de estreptococos do grupo mutans e imunoglobulinas anti-streptococcus mutans na saliva.. Rev. Odontol. UNESP.

[41] Victoria CG, Y Benguigui, FJ López Antuñano, G Schumunis, J Yvnes (1997). Infecciones respiratorias en niños..

[42] López BIM, Sepúlveda H, Valdés I (1997). Acute respiratory illnesses in the first 18 months of life.. Rev Panm Salud Publica.

[43] Di Francesco, RC, Passerotii G, Paulucci B, Miniti A (2004). Respiração oral na criança: repercussões diferentes de acordo com o diagnóstico.. Rev. Bras. Otorrinolaringol.

[44] Escobar AMU, Ogawa AR, Hiratsuka M, Kawashita MY, Teruya PY, Grisi S (2002). Aleitamento materno e condições socioeconômico-culturais: fatores que levam ao desmame precoce. Rev. Bras.. Saude. Mater. Infant.

[45] Kummer SC, Giugliani ERJ, Susin LO, Folletto JL, Lermen N R, Wu, VYJ (2000). Evolução do padrão de aleitamento materno.. Rev. Saúde Públ.

